# Identification of biological correlates associated with respiratory failure in COVID-19

**DOI:** 10.1186/s12920-020-00839-1

**Published:** 2020-12-11

**Authors:** Jung Hun Oh, Allen Tannenbaum, Joseph O. Deasy

**Affiliations:** 1 Department of Medical Physics, Memorial Sloan Kettering Cancer Center, New York, NY USA; 2 Departments of Computer Science and Applied Mathematics & Statistics, Stony Brook University, Stony Brook, NY USA

**Keywords:** COVID-19, SARS-CoV-2, Single-nucleotide polymorphisms, Genome-wide association study, Respiratory failure, Bioinformatics

## Abstract

**Background:**

Coronavirus disease 2019 (COVID-19) is a global public health concern. Recently, a genome-wide association study (GWAS) was performed with participants recruited from Italy and Spain by an international consortium group.

**Methods:**

Summary GWAS statistics for 1610 patients with COVID-19 respiratory failure and 2205 controls were downloaded. In the current study, we analyzed the summary statistics with the information of loci and *p*-values for 8,582,968 single-nucleotide polymorphisms (SNPs), using gene ontology analysis to determine the top biological processes implicated in respiratory failure in COVID-19 patients.

**Results:**

We considered the top 708 SNPs, using a *p*-value cutoff of 5 × 10^− 5^, which were mapped to the nearest genes, leading to 144 unique genes. The list of genes was input into a curated database to conduct gene ontology and protein-protein interaction (PPI) analyses. The top ranked biological processes were wound healing, epithelial structure maintenance, muscle system processes, and cardiac-relevant biological processes with a false discovery rate < 0.05. In the PPI analysis, the largest connected network consisted of 8 genes. Through a literature search, 7 out of the 8 gene products were found to be implicated in both pulmonary and cardiac diseases.

**Conclusion:**

Gene ontology and PPI analyses identified cardio-pulmonary processes that may partially explain the risk of respiratory failure in COVID-19 patients.

## Background

Coronavirus disease 2019 (COVID-19) caused by a novel coronavirus (severe acute respiratory syndrome coronavirus 2, SARS-CoV-2) has resulted in a global pandemic with a rapidly developing global health and economic crisis [1]. Most people with COVID-19 are asymptomatic or experience only mild symptoms [2]. However, about 5% of patients infected with the coronavirus develop acute lung injury and acute respiratory distress syndrome, possibly leading to lethal lung damage and even death [3].

The most common reported comorbidities associated with poor outcomes in COVID-19 include hypertension, diabetes, cardiovascular disease, and chronic respiratory infections [4, 5]. However, the underlying molecular mechanisms in severe COVID-19 and their interplay with such comorbidities or clinical factors are poorly understood [6].

To identify putative biomarkers that can help better understand the molecular basis of COVID-19, Blanco-Melo et al. investigated the host transcriptional response to SARS-CoV-2 and other respiratory infections through in vitro, ex vivo, and in vivo experiments [1]. Bioinformatical approaches including gene ontology and protein-protein interaction (PPI) analyses were performed to identify key biological correlates. To investigate key genetic variants associated with respiratory failure in COVID-19 patients, a genome-wide association study (GWAS) was carried out on participants recruited from Italy and Spain [7]. In the current study, we performed an in-depth biological characterization including gene ontology and PPI analyses on summary statistics that resulted from the GWAS analysis in order to identify key biological correlates relevant to respiratory failure in COVID-19 patients.

## Methods

The GWAS conducted by an international consortium group involved 1980 patients with severe acute respiratory failure induced by COVID-19 at seven hospitals in Italy and Spain [7]. After quality control, the final case-control cohort included 835 patients and 1255 control participants from Italy and 775 patients and 950 control participants from Spain. After genotyping and imputation on genome build GRCh38, univariate analysis was performed for 8,582,968 single-nucleotide polymorphisms (SNPs). The resulting summary statistics including individual SNP positions and *p*-values were submitted to the European Bioinformatics Institute (www.ebi.ac.uk/gwas; accession numbers, GCST90000255 and GCST90000256) and are available from www.c19-genetics.eu. The GCST90000255 was the main analysis in which all the association statistics were corrected for the top 10 principal components (PCs), whereas in the additional analysis of GCST90000256, association statistics were corrected for the top 10 PCs, age, and sex. In [7], the main results were found in the analysis on GCST90000255, and GCST90000256 was used for ancillary analysis. In the current study, we therefore focused on the summary statistics of GCST90000255 for further biological analysis because the analysis on GCST90000255 resulted in more plausible biological correlates likely associated with respiratory failure than those in GCST90000256.

To further enrich gene ontology terms with more plausible SNPs likely relevant to acute respiratory failure in COVID-19, we employed a relaxed *p*-value of 5 × 10^− 5^ as a filtering threshold on the summary statistics. The SNPs with *p*-values < 5 × 10^− 5^ were mapped to nearest genes using a 50 kb window on both upstream and downstream sides of each gene. The resulting list of genes was together fed into MetaCore software (Thompson Reuters, New York, NY) for gene ontology analysis. Further PPI analysis was performed to explore the largest connected network among the resulting list of genes with an option of ‘Direct interactions’ as a network building algorithm in MetaCore software, assuming that interacting proteins in a biological network may have the same or similar molecular functions [8–10].

To complement the biological interpretation using genes that were identified based on the proximity of candidate SNPs, the biological analysis described above was repeated using genes identified as expression quantitative trait loci (eQTL) targets from the Genotype-Tissue Expression (GTEx) database for tissues that appear to be relevant to respiratory failure, including the aorta, coronary artery, skeletal muscle, lung, and atrial appendage and left ventricle in the heart [11].

## Results

### Gene ontology analysis

In our analysis, with a *p*-value threshold of 5 × 10^− 5^ applied to the summary statistics, 708 SNPs remained and a corresponding set of 144 unique genes in autosomes was found (Additional file 1). The list of genes was fed into a MetaCore database. Table 1 shows the top 10 biological processes and corresponding genes that appear to be relevant to respiratory failure in COVID-19 patients, all with false discovery rate (FDR) values < 0.05. Wound healing, epithelial structure maintenance, muscle system process, and cardiac-relevant biological processes were top-ranked.

**Table 1:** The top 10 significant biological processes likely associated with respiratory failure in COVID-19 patients, using genes that were identified based on the proximity of 708 SNPs. The genes for each biological process belong to the list of 144 genes. FDR: false discovery rate

Ranking	Gene Ontology	FDR	Genes
1	Wound healing	1.962E-02	ADAMTS13, CCR9, CXCR6, DMBT1, EPPK1, GATA4, ITGB3, MYH1, MYH2, MYH4, PLEC, PRTN3, SMAD3, TFF1, TFF3, UBASH3A
2	Epithelial structure maintenance	1.962E-02	LDB2, TFF1, TFF2, TFF3
3	Cardiac ventricle development	1.962E-02	CCR9, CXCR6, GATA4, ID2, MYH1, MYH4, PTBP1, SLIT3, SUFU
4	Ventricular septum development	1.962E-02	CCR9, CXCR6, GATA4, ID2, SLIT3, SUFU
5	Cardiac septum development	1.962E-02	CCR9, CXCR6, GATA4, ID2, MYH1, MYH4, SLIT3, SUFU
6	Transdifferentiation	1.962E-02	GATA4, SMAD3
7	Muscle system process	2.106E-02	CCR9, CHRNA1, CXCR6, GATA4, MYH1, MYH2, MYH4, TMOD2, TMOD3
8	Cellular component maintenance	2.518E-02	CCR9, CXCR6, DLGAP1, ERC2, PRTN3, TANC1
9	Embryonic foregut morphogenesis	2.624E-02	GATA4, SMAD3
10	Response to virus	2.783E-02	AZU1, CCR9, CXCR6, DDX1, DMBT1, GATA4, IL12A, TRIM5, TRIM6, TRIM22, TRIM34

### PPI network analysis

**Fig. 1:**
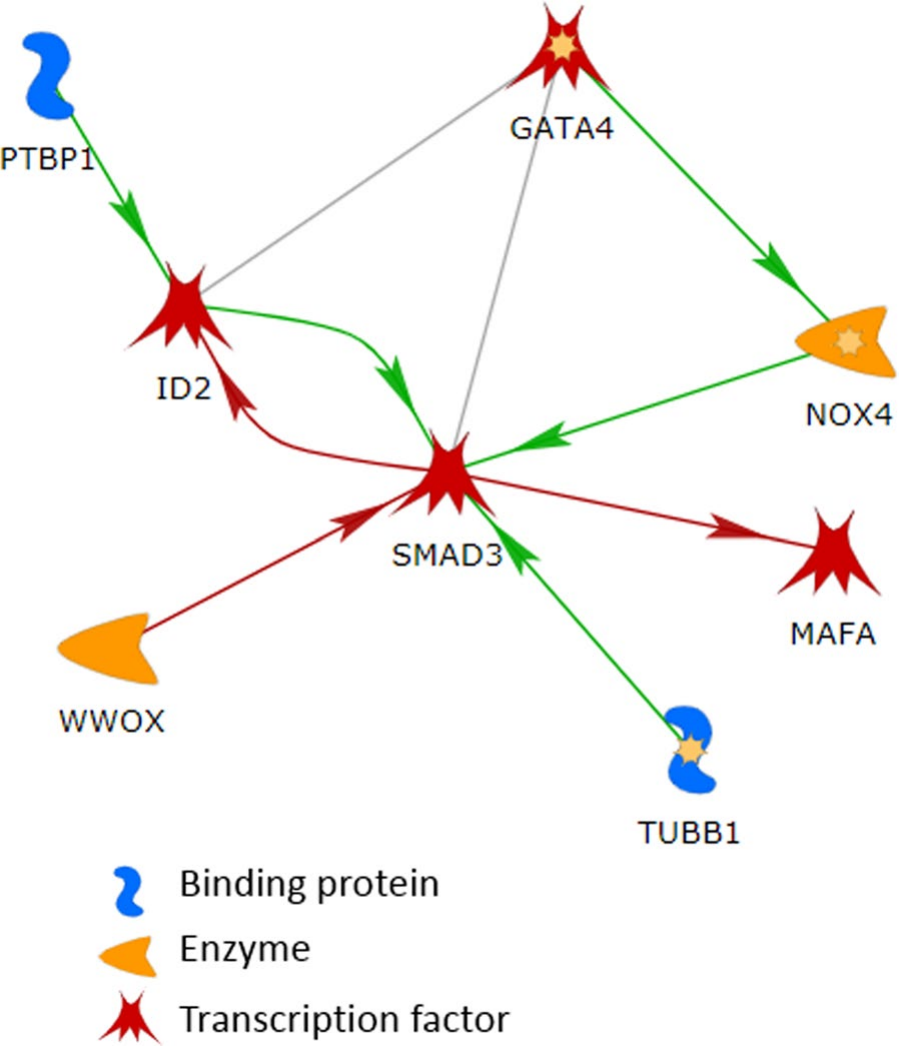
The largest connected network in the list of 144 genes. The line colors indicate the activation (green), inhibition (red), and unspecified (gray) effects

The largest connected PPI network in the list of 144 genes is shown in Fig. 1. The PPI network consisted of 8 gene products for the following genes: *GATA4*, *ID2*, *MAFA*, *NOX4*, *PTBP1*, *SMAD3*, *TUBB1*, and *WWOX*. We conducted a literature search in PubMed to investigate the potential associations between those 8 genes/proteins and pulmonary or cardiac diseases. Additional file 2 contains a table that lists an overview of reported studies in terms of the associations. Interestingly, except for *MAFA* that is involved in insulin secretion, all 7 gene products were found to be implicated in both pulmonary and cardiac diseases.

### eQTL analysis

Tissue-specific genes that had significant associations with the 708 SNPs were identified from GTEx V8 [11]. Six tissues were examined including the aorta, coronary artery, skeletal muscle, lung, and atrial appendage and left ventricle in the heart, resulting in 17, 4, 21, 17, 8, and 10 genes, respectively (Additional file 3). Gene ontology and PPI analyses described above were repeated using the resultant 34 unique genes. Among 34 gene products, no any interaction was found. Table 2 shows the top 10 biological processes and corresponding genes, all with FDR values < 0.05. All biological processes were involved in 3 gene products: *CCR3*, *CCR5*, and *CXCR6*. Chemokine-related biological processes were top-ranked.

**Table 2:** The top 10 significant biological processes likely associated with respiratory failure in COVID-19 patients, using genes identified as expression quantitative trait loci targets from 6 tissues for 708 SNPs. The genes for each biological process belong to the list of 34 genes. FDR: false discovery rate

Ranking	Gene Ontology	FDR	Genes
1	Angioblast cell migration	3.293E-03	CCR3, CCR5, CXCR6
2	Chemokine-mediated signaling pathway	3.946E-03	CCR3, CCR5, CXCR6
3	Cellular response to chemokine	3.946E-03	CCR3, CCR5, CXCR6
4	Response to chemokine	3.946E-03	CCR3, CCR5, CXCR6
5	Positive regulation of apoptotic process by virus	3.946E-03	CCR3, CCR5, CXCR6
6	Positive regulation by symbiont of host apoptotic process	4.926E-03	CCR3, CCR5, CXCR6
7	Positive regulation by symbiont of host programmed cell death	4.926E-03	CCR3, CCR5, CXCR6
8	Killing by symbiont of host cells	4.926E-03	CCR3, CCR5, CXCR6
9	Modulation by virus of host apoptotic process	4.926E-03	CCR3, CCR5, CXCR6
10	Positive regulation by organism of programmed cell death in other organism involved in symbiotic interaction	4.926E-03	CCR3, CCR5, CXCR6

Among the 708 SNPs, 41, 48, 5, 59, and 180 SNPs had eQTL associations for multiple tissues with 6, 5, 4, 3, and 2 tissues, respectively (Additional file 4). In addition, rs8093548 (chr18, pos: 79876451 in GRCh38), rs4799099 (chr18, pos: 79879585), and rs4799100 (chr18: pos: 79880207) had eQTL associations with the most genes; all three SNPs had eQTL associations with the same five genes – *HSBP1L1*, *PQLC1*, *RBFA*, *RBFADN*, and *TXNL4A* – in five tissues including the aorta, skeletal muscle, lung, and atrial appendage and left ventricle in the heart.

## Discussion

Summary statistics from a GWAS dataset for respiratory failure in COVID-19 patients were analyzed employing bioinformatics techniques. To enrich the biological discovery, a relaxed *p*-value of 5 × 10^− 5^ was adopted, which likely enabled the inclusion of much potential genomic information in the analysis and the identification of plausible biological correlates associated with pulmonary or cardiac symptoms. A list of SNPs filtered by the relaxed p-value threshold was mapped to nearby genes. The resulting 144 genes were fed into a MetaCore database for gene ontology and PPI analyses.

Gene ontology analysis identified wound healing, cardiac-related biological process, and muscle system process as key correlates. For PPI analysis, we attempted to find the largest connected network in the list of 144 genes, assuming that interacting proteins in a biological network tend to have the same or similar molecular functions. As a result, the largest connected network consisted of 8 gene products from the following genes: *GATA4*, *ID2*, *MAFA*, *NOX4*, *PTBP1*, *SMAD3*, *TUBB1*, and *WWOX*. A literature search was conducted through PubMed to investigate whether there are previously reported results in terms of biological associations between these genes/proteins and respiratory or cardiac symptoms. Interestingly, we found that most of these gene products are relevant to both respiratory and cardiac diseases. In what follows, we describe the role of these biomarkers in biology.

A study reported that *GATA4* plays a critical role as a transcription factor in the normal pulmonary development [12]. *GATA4* also has been found to be a human candidate gene relevant to congenital heart disease [13, 14]. Several studies showed that *GATA4* is a key protein responsible for the development of the lung, heart, and diaphragm in mice [15–17].

Arwood et al. described a mechanism of pulmonary hypertension in heart failure with preserved ejection fraction (HFpEF), using transcriptome-wide RNA sequencing [18]. When comparing the transcriptomic difference between patients without pulmonary hypertension and those with combined post- and pre-capillary pulmonary hypertension, six differentially expressed genes were identified. In a further replication test on an independent cohort, only *ID2* was validated and in an additional animal study, *ID2* expression was significantly upregulated in mice with HFpEF and pulmonary hypertension compared to control mice. Another study showed a functional role of *ID2* as one of the culprit genes in both the arterial and the venous poles of the heart [19].

An increased expression of *NOX4* and *TGF-β* was found to be correlated with the increased volume in both airway smooth muscle mass and epithelial cells of small airways in patients with chronic obstructive pulmonary disease (COPD) [20]. Another study reported that the upregulation of *NOX4* in the heart induced cardiac remodeling, suggesting its potential role to reduce the severity of established heart failure [21].

Gauldie et al. demonstrated a cascade of biological interactions among inflammation, *TGF-β* activation, *SMAD3* signaling, pulmonary fibrosis, and emphysema [22]. Huang et al. found that *SMAD3* is a key mediator in chronic cardiovascular disease, and plays a critical role in hypertensive cardiac remodeling [23].

At 4 and 24 h after respiratory syncytial virus infection, gene expression profiles in human bronchial epithelial cells were analyzed [24]. Among the six genes that were associated with respiratory disease and were significantly altered at both 4 and 24 h post-infection, *TUBB1* was the only gene observed to be downregulated at both time points. Freson et al. showed that the *TUBB1* Q43P functional variant may be a protective genetic factor against cardiovascular disease [25].

Caruso et al. observed the downregulation of *miR-124* in patients with pulmonary arterial hypertension and its central role in contributing to abnormal cell proliferation via *PTBP1* and *PKM2* [26]. Recently, Fochi et al. showed the emerging role of *RBM20* and *PTBP1* as key splicing factors in heart development and cardiovascular disease [27].

A study reported that the loss of *WWOX* promoted cell proliferation in pulmonary artery smooth muscle cells and contributed to pulmonary vascular remodeling in pulmonary arterial hypertension [28]. Another study reported the vital implications of *WWOX* in atherosclerosis and cardiovascular diseases [29].

*MAFA* has not been found to be directly related to pulmonary or cardiac symptoms in the literature review. However, *MAFA* has been shown to be a key regulator that controls genes implicated in insulin secretion [30, 31]. A recent study indicated that a number of patients with COVID-19, who were comorbid with diabetes or diabetes-related traits, had increased *ACE2* expression [32]. This suggests that *ACE2* appears to be a potentially key molecular link between insulin resistance and COVID-19 severity [33].

The combined evidence indicated that lung disease is likely to be associated with cardiovascular risk. Further research should be warranted to identify the common biological processes between lung and heart diseases and the interplay between them.

We further assessed various filtering thresholds. With a stricter *p*-value of 1 × 10^− 5^, 390 SNPs and corresponding 27 unique genes in autosomes remained. Gene ontology analysis with a relatively small number of genes resulted in immunity-related biological processes as the top important covariates. The top two biological processes were chemokine-mediated signaling pathway (FDR = 2.906E-3) and CD8-positive, gamma-delta intraepithelial T cell differentiation (FDR = 2.906E-3). With a more relaxed p-value of 1 × 10^− 4^, 1112 SNPs and corresponding 243 unique genes in autosomes remained. Gene ontology analysis with those genes resulted in biological processes that are irrelevant to respiratory failure, which is likely due to false positives added in the analysis. This implies that the selection of an optimal threshold is critical to identify real biological correlates. Information informed by machine learning-based predictive modeling on GWAS data, which we employed in other studies [8, 9], can help resolve the issue.

Biological analyses using genes that were identified based on the proximity of candidate SNPs resulted in cardio-pulmonary processes as associated with respiratory failure. In particular, 7 out of the 8 gene products in the largest connected network were found to be implicated in both pulmonary and cardiac diseases. In contrast, the selection of genes identified as eQTL targets uncovered chemokine-related biological processes, indicating the association with the immune system. This suggests that an integrated analysis of the two methods in identifying relevant genes can help better understand the underlying biological mechanisms of respiratory failure in COVID-19 patients.

## Conclusions

We analyzed summary statistics from a GWAS dataset where individual SNPs were tested for associations with respiratory failure in COVID-19 patients. Bioinformatics approaches with SNPs filtered using a relaxed *p*-value enabled the identification of plausible biological correlates that are likely to be relevant to pulmonary or cardiac symptoms. When genotyping data become available, a more in-depth analysis using machine learning and bioinformatics techniques will provide greater insights into the underlying mechanisms of respiratory failure in COVID-19 patients.

## Supplementary Information

**Additional file 1.** 144 genes identified based on the proximity of 708 candidate SNPs.

**Additional file 2.** An overview of reported studies in terms of the associations between genes/proteins and pulmonary or cardiac symptoms.

**Additional file 3.** For 708 candidate SNPs, eQTL information obtained from GTEx V8 for 6 tissues, including the aorta, coronary artery, skeletal muscle, lung, and atrial appendage and left ventricle in the heart.

**Additional file 4.** SNPs with eQTL associations in multiple tissues.

## Data Availability

All the data analyzed in this study are available at http://www.c19-genetics.eu.

## Abbreviations

COVID-19: Coronavirus disease 2019
GWAS: Genome-wide association study
SNP: Single-nucleotide polymorphism
PPI: Protein-protein interaction
PC: Principal component
FDR: False discovery rate
HFpEF: Heart failure with preserved ejection fraction
COPD: Chronic obstructive pulmonary disease
eQTL: Expression quantitative trait loci
GTEx: Genotype-Tissue Expression
