# Supplementary material for: Identification of biological correlates associated with respiratory failure in COVID-19
Source: BMC Med Genomics. 2020 Dec 11;13:186. doi: 10.1186/s12920-020-00839-1 (PMC7729705; doi:10.1186/s12920-020-00839-1)
Supplement: Supplementary file 2 — Additional file 2. An overview of reported studies in terms of the associations between genes/proteins and pulmonary or cardiac symptoms. [file 12920_2020_839_MOESM2_ESM.docx]

**Table.** An overview of reported studies in terms of the associations between genes/proteins and pulmonary or cardiac symptoms. The MAFA gene is involved in insulin secretion.

|  | Pulmonary symptoms | Cardiac symptoms | Insulin |
| --- | --- | --- | --- |
| GATA4 | Ackerman [1] | Garg [2]  Sarkozy [3] |  |
| ID2 | Arwood [4] | Jongbloed [5] |  |
| NOX4 | Guo [6] | Zhao [7] |  |
| SMAD3 | Gauldie [8] | Huang [9] |  |
| TUBB1 | Huang [10] | Freson [11] |  |
| PTBP1 | Caruso [12] | Fochi [13] |  |
| WWOX | Teixeira Gomes [14] | Tanna [15] |  |
| MAFA |  |  | Wang [16]  Zhang [17] |

**References**

1. Ackerman KG, Wang J, Luo L, Fujiwara Y, Orkin SH, Beier DR: **Gata4 is necessary for normal pulmonary lobar development**. *Am J Respir Cell Mol Biol* 2007, **36**(4):391-397.

2. Garg V, Kathiriya IS, Barnes R, Schluterman MK, King IN, Butler CA, Rothrock CR, Eapen RS, Hirayama-Yamada K, Joo K *et al*: **GATA4 mutations cause human congenital heart defects and reveal an interaction with TBX5**. *Nature* 2003, **424**(6947):443-447.

3. Sarkozy A, Conti E, Neri C, D'Agostino R, Digilio MC, Esposito G, Toscano A, Marino B, Pizzuti A, Dallapiccola B: **Spectrum of atrial septal defects associated with mutations of NKX2.5 and GATA4 transcription factors**. *J Med Genet* 2005, **42**(2):e16.

4. Arwood MJ, Vahabi N, Lteif C, Sharma RK, Machado RF, Duarte JD: **Transcriptome-wide analysis associates ID2 expression with combined pre- and post-capillary pulmonary hypertension**. *Sci Rep* 2019, **9**(1):19572.

5. Jongbloed MR, Vicente-Steijn R, Douglas YL, Wisse LJ, Mori K, Yokota Y, Bartelings MM, Schalij MJ, Mahtab EA, Poelmann RE *et al*: **Expression of Id2 in the second heart field and cardiac defects in Id2 knock-out mice**. *Dev Dyn* 2011, **240**(11):2561-2577.

6. Guo X, Fan Y, Cui J, Hao B, Zhu L, Sun X, He J, Yang J, Dong J, Wang Y *et al*: **NOX4 expression and distal arteriolar remodeling correlate with pulmonary hypertension in COPD**. *BMC Pulm Med* 2018, **18**(1):111.

7. Zhao QD, Viswanadhapalli S, Williams P, Shi Q, Tan C, Yi X, Bhandari B, Abboud HE: **NADPH oxidase 4 induces cardiac fibrosis and hypertrophy through activating Akt/mTOR and NFκB signaling pathways**. *Circulation* 2015, **131**(7):643-655.

8. Gauldie J, Kolb M, Ask K, Martin G, Bonniaud P, Warburton D: **Smad3 signaling involved in pulmonary fibrosis and emphysema**. *Proc Am Thorac Soc* 2006, **3**(8):696-702.

9. Huang XR, Chung AC, Yang F, Yue W, Deng C, Lau CP, Tse HF, Lan HY: **Smad3 mediates cardiac inflammation and fibrosis in angiotensin II-induced hypertensive cardiac remodeling**. *Hypertension* 2010, **55**(5):1165-1171.

10. Huang YC, Li Z, Hyseni X, Schmitt M, Devlin RB, Karoly ED, Soukup JM: **Identification of gene biomarkers for respiratory syncytial virus infection in a bronchial epithelial cell line**. *Genomic Med* 2008, **2**(3-4):113-125.

11. Freson K, De Vos R, Wittevrongel C, Thys C, Defoor J, Vanhees L, Vermylen J, Peerlinck K, Van Geet C: **The TUBB1 Q43P functional polymorphism reduces the risk of cardiovascular disease in men by modulating platelet function and structure**. *Blood* 2005, **106**(7):2356-2362.

12. Caruso P, Dunmore BJ, Schlosser K, Schoors S, Dos Santos C, Perez-Iratxeta C, Lavoie JR, Zhang H, Long L, Flockton AR *et al*: **Identification of MicroRNA-124 as a Major Regulator of Enhanced Endothelial Cell Glycolysis in Pulmonary Arterial Hypertension via PTBP1 (Polypyrimidine Tract Binding Protein) and Pyruvate Kinase M2**. *Circulation* 2017, **136**(25):2451-2467.

13. Fochi S, Lorenzi P, Galasso M, Stefani C, Trabetti E, Zipeto D, Romanelli MG: **The Emerging Role of the RBM20 and PTBP1 Ribonucleoproteins in Heart Development and Cardiovascular Diseases**. *Genes (Basel)* 2020, **11**(4):402.

14. Teixeira Gomes M, Chen J, Haider S, Bai Y, Singla S, Machado RF: **Smooth Muscle Cell Loss of the Tumor Suppressor WWOX Contributes to the Development of Pulmonary Hypertension**. *Am J Respir Crit Care Med* 2020, **201**:A7211.

15. Tanna M, Aqeilan RI: **Modeling WWOX Loss of Function in vivo: What Have We Learned?** *Front Oncol* 2018, **8**:420.

16. Wang H, Brun T, Kataoka K, Sharma AJ, Wollheim CB: **MAFA controls genes implicated in insulin biosynthesis and secretion**. *Diabetologia* 2007, **50**(2):348-358.

17. Zhang C, Moriguchi T, Kajihara M, Esaki R, Harada A, Shimohata H, Oishi H, Hamada M, Morito N, Hasegawa K *et al*: **MafA is a key regulator of glucose-stimulated insulin secretion**. *Mol Cell Biol* 2005, **25**(12):4969-4976.
